# Risk and protective factors for recovery at 3-year follow-up after first-episode psychosis onset: a multivariate outcome approach

**DOI:** 10.1007/s00127-023-02579-w

**Published:** 2023-10-20

**Authors:** Clara Serra-Arumí, Philippe Golay, Vincent Bonnarel, Livia Alerci, Lilith Abrahamyan Empson, Philippe Conus, Luis Alameda

**Affiliations:** 1 Etiopathogenesis and Treatment of Severe Mental Disorders (MERITT), Teaching, Research and Innovation Unit, Institut de Recerca Sant Joan de Déu, Esplugues de Llobregat, Spain; 2 Parc Sanitari Sant Joan de Déu, Sant Boi de Llobregat, Spain; 3 Universitat de Barcelona, Barcelona, Spain; 4 Department of Psychiatry, General Psychiatry Service, Treatment and Early Intervention in Psychosis Program, Centre Hospitalier Universitaire Vaudois (CHUV), Place Chauderon, 18, 1003 Lausanne, Switzerland; 5 Department of Psychosis Studies, Institute of Psychiatry, Psychology and Neuroscience, King’s College London, London, UK; 6 Centro de Investigación Biomédica en Red de Salud Mental (CIBERSAM), Departamento de Psiquiatría, Instituto de Biomedicina de Sevilla (IBIS), Hospital Universitario Virgen del Rocío, Universidad de Sevilla, Seville, Spain

**Keywords:** Early intervention, Early psychosis, Recovery, Outcome, Protective factors

## Abstract

**Purpose:**

Recovery in people with first-episode psychosis (FEP) remains a major issue. When risk factors are studied in relation to the disorder, potential protective factors should also be considered since they can modulate this relationship. This study is aimed at exploring which premorbid and baseline characteristics are associated with a good and poor global recovery in patients with FEP at 3-year follow-up.

**Methods:**

We categorized patients’ outcome by using a Latent Class Analysis (LCA) considering a multimodal set of symptomatic and functional outcomes. A Mixed effects Models Repeated Measures analysis of variance (MMRM) was used to highlight group differences over time on symptomatic and functional outcomes assessed during the 3-year follow-up.

**Results:**

A total of 325 patients with FEP aged between 18 and 35 years were included. Two groups were identified. A total of 187 patients (57.5%) did not achieve recovery, and 138 patients (42.5%) achieved recovery. Recovered patients had generally a better premorbid and baseline profile in comparison with non-recovered patients (as among which shorter duration of untreated psychosis (DUP), higher degree of insight, better functional level and lower illness severity at baseline). The trajectories for the psychopathological and functional outcomes over 36 months differed between the non-recovered and the recovered group of patients.

**Conclusions:**

Our results pointed to some variables associated with recovery, acting as potential protective factors. These should be considered for early intervention programs to promote psychological resilience specifically in those with a worse prognosis in order to mitigate the effects of the variables that make them more vulnerable to poorer outcome.

## Introduction

Protective factors (PF) are defined as characteristics (biological, psychological, family, or community) and resources that are associated with increased likelihood of positive outcomes and a reduced likelihood of negative impact from exposure to risk factors (RF) [1]. These factors favor one’s ability to cope, adapt and recover when facing stressful situations [2]. Over the last decades, PF has been increasingly studied in relation to mental health and mental disorders, including psychotic disorders [3–5].

While research in psychiatry often places an emphasis on RF of disorder, which stem from the epidemiological literature and are essential to understanding of etiopathogenesis, PF are often overlooked, even though they can modulate and influence this relationship [2, 6]. Furthermore, PF should be differentiated from RF, since there can be non-reciprocal PF to the risk variables, which go beyond being complementary factors, and are specific personal, psychosocial or environmental characteristics that can improve the likelihood of positive outcomes and also reduce the effects of the risk on this final result [4, 7]. Identifying these protective variables can be helpful to detect which profiles of premorbid and baseline sociodemographic and clinical characteristics are associated with being protective against a poor prognosis [6, 8]. Focusing on these may provide important insights into the understanding of positive trajectories and conditions that are associated with a good recovery in people with first-episode psychosis (FEP). Moreover, the identification of these protective characteristics of poor outcomes may be relevant in developing and implementing early interventions focused on those troublesome areas for timely recovery achievement in people with FEP [9].

Understanding which factors protect against poor outcomes of recovery would expand the knowledge on what variables help build and raise resilience in people with FEP, since individual competences and environmental factors can be associated with positive outcomes, even in the context of adversity [10, 11]. Resilience involves a developmental progression [12] as new vulnerabilities and strengths may emerge from changing life circumstances encompassing positive adaptation in the context of significant adversity [13].

Furthermore, resilience and protective personal resources are associated with recovery in psychotic disorders, emphasizing that resilience is modifiable and that patients could improve if it is strengthened [14]. Moreover, modifiable and dynamic PF are relevant to clinical practice as they can be targeted by recovery-oriented early interventions in people with FEP [9], which is a promising avenue of research for recovery. Identifying early PF may help clinicians provide aims for treating other patients that present predominant RF and set standards in terms of early intervention; monitor how these early RF evolve and how more PF develop early in the treatment. In addition, recognizing early PF is crucial for designing preventive interventions for developing psychosis in people at high risk with several risk characteristics that make them more vulnerable to psychosis.

So far, few studies in FEP have used the Latent Class Analysis (LCA) to classify patients and identify recovery/non-recovery or symptomatic remission/non-remission groups or trajectories [15–18]. This is a robust methodological approach that would improve the strategy adopted hitherto to determine the groups of patients according to their recovery status, using a multimodal measure of positive outcomes as a criterion, rather than determining the good or bad outcomes based on very narrow and clear-cut predefined thresholds (such as total Global Assessment of Functioning (GAF) score above 60 [19]). There is growing evidence that using a multimodal set of variables including aspects of the context as well as clinical domains may be more useful than an algorithmic categorization based on predefined cutoffs on a single item [20]. Therefore, LCA using a multimodal set of variables allows for a more comprehensive definition of positive outcome.

Based on the above, this study aimed at exploring which premorbid and baseline sociodemographic and clinical characteristics are associated with a good global recovery in patients with FEP at 3-year follow-up. We categorized patients by using a LCA considering a multimodal set of relevant clinical and contextual variables to determine good and bad outcomes. We also compared symptomatic and functioning 3-year trajectories in order to highlight when good and bad outcomes become evident, and specifically, to detect which premorbid and baseline characteristics are protective predictors in the first 3 years of treatment after a FEP. Results will be discussed not only with a focus on RF but providing an insight into the PF too and the related implications in early intervention.

## Material and methods

### Participants and procedure

The sample included patients that were enrolled for 3 years in the Treatment and early Intervention in Psychosis Program (TIPP), a specialized early psychosis (EP) program run by Lausanne University Hospital's Department of Psychiatry, in Switzerland [21]. To be admitted into the program, patients must meet the following inclusion criteria: age from 18 to 35, living in the hospital's catchment area, and meeting the criteria for psychosis as defined by the ‘psychosis threshold’ subscale in the Comprehensive Assessment of At-Risk Mental States (CAARMS) instrument [22]. Patients with organic brain disease, an intelligence quotient (IQ) under 70, or those on antipsychotic medication for more than six months are referred to other programs.

All patients treated within the TIPP are fully assessed at baseline, after 2 months, 6 months and then prospectively every 6 months to monitor symptomatic and functional outcomes, comorbidities, contextual aspects and treatments.

In the TIPP program, each patient is followed up by a psychiatrist and a case manager. The program offers an integrated biopsychosocial treatment based on psychotherapy, psychoeducation, family support, cognitive assessment and remediation (when needed), social support, assistance in finding employment, psychological interventions for cannabis use, and pharmacological treatment. A specially designed questionnaire (TIPP Initial Assessment Tool (TIAT; available online) [23] is completed for all patients enrolled in the program by their case managers who have up to 100 instances of contact with patients during the 3 years of treatment. It assesses demographic characteristics, past medical history, exposure to life events as well as symptoms and functioning. It is completed on the basis of information gathered from patients and their family over the first few weeks of treatment and can be updated during follow-up if new information emerges. Follow-up assessments, exploring various aspects of psychopathology, are conducted by a research psychologist (for psychopathology) after 2, 6, 12, 18, 24, 30, and 36 months of treatment [21]. This study was approved by the Human Research Ethics Committee of the Canton Vaud (protocol #2020–00272). The data generated by the follow-up of all patients were used in the study on the basis of their informed consent. This is a prospective study based on the first 329 patients who completed 36-months follow-up and for whom data on trauma and psychopathology were available.

### Measures

#### Premorbid and baseline variables

The following scales were administered: the Premorbid Adjustment Scale (PAS) [24], the Global Assessment of Functioning (GAF) [25], the Social and Occupational Functioning Assessment Scale (SOFAS) [26], and the Clinical Global Impression (CGI) [27]. A broad range of potential PF were considered based on meta-analytical evidence showing their association with recovery. Being female, being enrolled in education or a job, having a shorter DUP, a shorter duration of untreated illness (DUI), a better premorbid adjustment prior to the FEP onset, a lower severity of symptoms (positive and negative) at baseline, and a better cognitive functioning at baseline are associated with achieving recovery [9, 28].

#### Multimodal operationalization of positive (recovered) versus negative (non-recovered) outcomes at discharge

To operationalize the composite measure of recovery at discharge, six clinical, functional and contextual variables previously used to define recovery in patients with psychosis [29–32] and having been identified as important determinants of recovery in the TIPP sample [33, 34], were selected from the available assessments, and subsequently included in the LCA (see ‘Statistical analysis’ section). This led to creating a multimodal measure of positive outcomes at 3-year follow-up. These variables were (i) Symptomatic response. The Andreasen criteria [35] based on the last Positive and Negative Syndrome Scale (PANSS) [36] assessment was used to determine whether patients achieved symptomatic response or not over the last year of follow-up. PANSS assesses the severity of the psychotic symptoms through 30 items, scored from 1 (absent) to 7 (extreme). The criteria established by the Remission in Schizophrenia Working Group (RSWG) involve scoring mild or lower (≤ 3) severity on the following items: delusion (P1), unusual thought content (G9), hallucinatory behavior (P3), conceptual disorganization (P2), mannerisms/posturing (G5), blunted affect (N1), social withdrawal (N4), and lack of spontaneity (N6); (ii) Depression status. The Montgomery-Asberg Depression Rating Scale (MADRS) [37] was used to detect non-depressed and depressed patients. It is a 10-item scale, and each item yields a score of 0 to 6. Data from the last assessment available in the last year follow-up was utilized. The cutoff to identify non-depressed patients was under 10 in the total score [38]. Depressive symptoms were an important predictor of outcome in TIPP previous studies [34]; (iii) Insight level. Insight regarding the illness was categorized as complete, partial, or absent [39]. For the analysis, this variable was dichotomized into fully aware of the disorder or not. Previous analyses in TIPP showed its importance in patients’ recovery [33]; (iv) Functional level. The Social and Occupational Functioning Assessment Scale (SOFAS) was administered to provide a measure about social and occupational functioning [26]. The cutoff to detect patients with good functioning at 36-months follow-up was established at 60 or above, on a scale of 0 to 100 points; (v) Independent living. According to the Modified Location Code Index (MLCI), this included being the head of household, living alone, with partner, or with peers, and living with family with minimal supervision [40]; and, (vi) Occupational status. According to the Modified Vocational Status Index (MVSI), this included being paid or unpaid in full or part-time employment, an active student in school or university, head of household with employed partner (homemaker), and full or part-time volunteer [40].

#### Psychopathological and functional measures after 2, 6, 12, 18, 24, and 36-months follow-up

Psychopathology and functional level were scored at each assessment with the Positive and Negative Syndrome Scale (PANSS) [36], the Montgomery-Asberg Depression Rating Scale (MADRS) [37], the Global Assessment of Functioning (GAF) [25], and the Social and Occupational Functioning Assessment Scale (SOFAS) [26].

Symptomatic remission at the end of the program was defined by the last Positive and Negative Syndrome Scale assessment score in the last year of the program, following Andreasen criteria for remission (mild or lower (≤ 3) score on the following items: delusion, unusual thought content, hallucinatory behavior, conceptual disorganization, mannerisms, blunted affect, social withdrawal and lack of spontaneity; [35]).

### Statistical analysis

Latent Class Analysis (LCA) is a statistical method by which individuals can be classified into several groups, or latent classes, based on their pattern of answers on a set of categorical indicator variables. This allowed us to identify distinct patient recovery profiles focused on an outcomes-centered approach. The number of latent classes was determined by analyzing models including one to four classes. The six binary variables used to identify latent classes were: symptomatic response (PANSS), depression status (MADRS), insight level, functional level (SOFAS), occupational status (MVSI), and independent living (MLCI). We ensured data from at least four or more outcomes were available. The model parameters were obtained via the maximum likelihood estimates (MLE) of the conditional response probabilities. The optimal number of latent classes was based on the Bayesian Information Criterion (BIC) which highlighted a two classes solution (global recovery/global non-recovery) as the most preferable model.

To compare the two groups that emerged as latent classes from the LCA in terms of a broad range of potential RF and PF and in terms of functional and psychopathological trajectories, we performed a series of logistic regressions with global non-recovery/recovery groups as the dependent variable, and the baseline and follow-up data as predictors. Odds ratios (OR) were reported. For every unit increase in the independent variable, the odds of the dependent variable equaling a case is multiplied by the OR.

Mixed effects Models Repeated Measures analysis of variance (MMRM) was used to detect group differences over time on some different outcome measures assessed during follow-up. Time was introduced as a within-group factor and good outcomes as a between-group factor. For the model, the main effects for good-outcomes and time as well as their interaction were examined. Being interested in contrasting changes from the first assessment to the different follow-up assessments, and considering the numerous available measurements, planned comparisons within the MMRM were performed. This allowed us to examine differences between groups concerning rate of improvement in symptoms and functioning from the first assessment to the follow-up assessments (2, 6, 12, 18, 24, 30 and 36 months). The Akaike Information Criterion (AIC) coefficient was checked to determine the optimal within-subject covariance matrix. The following structures were tested: unstructured, autoregressive, compound-symmetric and Toeplitz as well as their heterogeneous versions.

MMRM is advantageous and differs from traditional repeated measures models, ANOVA and ANCOVA, in that all existing data is included in the model. MMRM does not require the imputation of missing data with hypothetical values. This approach relies on data being missing at random. Another advantage of MMRM is that the correlation of the repeated data on all occasions can be modeled (e.g., using heterogenous Toeplitz covariance).

The Latent Class Analysis (LCA) was conducted with MPlus version 8.3. Descriptive analyses and the comparison between groups were performed with SPSS version 26.0. All statistical tests were two-tailed, and statistical significance was established at alpha = 0.05.

## Results

### Recovered and non-recovered patients based on Latent Class Analysis

**Fig. 1:**
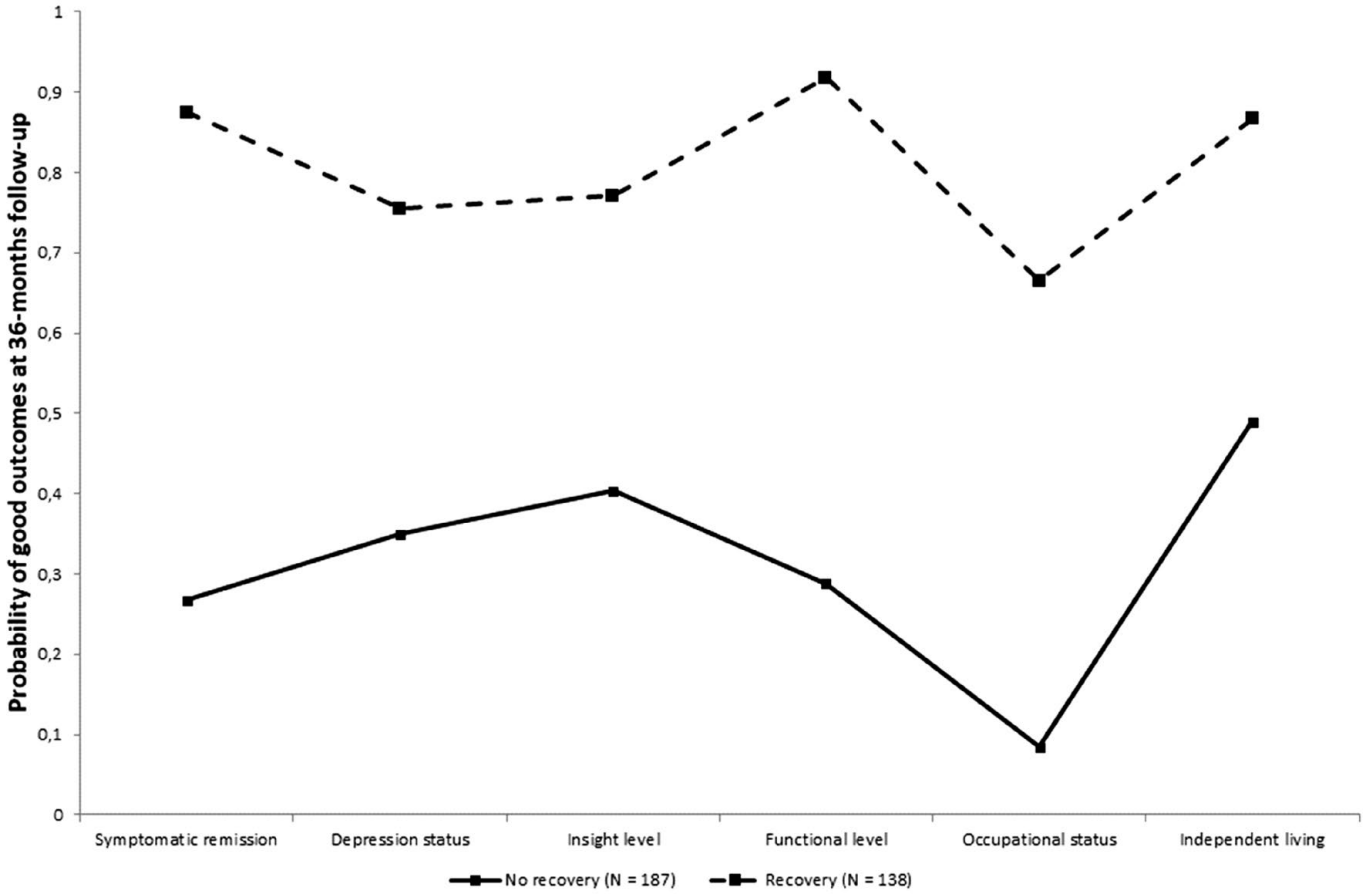
Probability of good outcomes at 36-months follow-up: identification of the non-recovered group and the recovered group of patients

In Table 1 is unveiled the LCA results including models from 1 to 4 classes. The sample included 325 patients. The BIC suggested the best LCA model had two latent classes. Their interpretation was straightforward: the non-recovered group with 187 patients (57.5%), and the recovered group with 138 patients (42.5%). Other less preferable statistical solution did not suggest alternative meaningful clustering. Figure 1 details the six variables collected at 3-year follow-up which build the global multivariate measure of positive outcomes and shows the clear identification of the two latent classes, which are the non-recovered group and the recovered group of patients, with their probability of good outcomes at 36-months follow-up.

**Table 1:** Latent Class Analysis results

Number of classes	1	2	3	4
BIC	2269.19	2132.42	2162.62	2195.34
Entropy	–	0.674	0.603	0.664
Size of the smallest class *N* (%)	325 (100.0)	138 (42.46)	53 (16.31)	14 (4.31)

*BIC* Bayesian Information Criterion, *N* number of participants; % = percentage

In order to further validate the proposed new multimodal criteria for recovery, we verified the proportion of patients that met Andreasen criteria for remission at the last assessment available in the last year of the TIPP program. We found a significant difference between the groups (*χ*^2^(1) = 76.482, *p* < 0.001), because 91.9% of the patients in the recovered group indeed met Andreasen criteria, while 75.0% of the patients in the non-recovered group were not in remission considering the Andreasen criteria.

### Description of the sample and comparison between recovered and non-recovered TIPP patients

The characteristics of the 325 early psychosis patients that did not achieve recovery and those who did at 36-months follow-up are summarized in Table 2. The mean age in years in the non-recovered group was 24.23, and in the recovered group 25.25 (odds ratio (OR) = 1.046, *p* = 0.059). Regarding sex, 72.2% of the non-recovered patients with FEP were male, and of the recovered group 63.0% (OR = 1.522, *p* = 0.081). Non-recovered patients had a longer DUP compared to recovered participants (OR = 0.597, *p* < 0.001). The participants from the non-recovered group were more likely to be hospitalized more than one time (54% of the non-recovered and 27% of the recovered), while being admitted just once was more common in the recovered group (27% of the non-recovered and 41% of the recovered) (OR = 0.529, *p* < 0.001). Non-recovered people were younger at the onset of the disorder (OR = 1.067, *p* = 0.005) and had fewer years of education (OR = 1.155, *p* = 0.005) in comparison with recovered participants. The non-recovered sample were less likely to be students (OR = 3.321, *p* < 0.001) or to have a full-time job (OR = 2.755, *p* = 0.011). Also, their unemployment rates were higher (57.1%) than in the recovered sample (36.0%). Non-recovered participants were more likely to live in a pension or care home (OR = 0.191, *p* = 0.044) or unsettled (OR = 0.246, *p* = 0.023), and rates of people living with family were lower (16.9%) than in recovered people (26.7%). Non-recovered patients had worse premorbid adjustment scores in all domains, childhood (OR = 0.099, *p* = 0.001), early adolescence (OR = 0.128, *p* = 0.006), social (OR = 0.258, *p* = 0.027), academic (OR = 0.141, *p* = 0.002), and overall (OR = 0.079, *p* = 0.002) in contrast with the recovered participants. Non-recovered people were more likely to have a forensic history (OR = 0.386, *p* = 0.013), and criminal offences during TIPP treatment (OR = 0.247, *p* = 0.017) than recovered people. Non-recovered group had higher rates of familial psychiatric history (OR = 0.535, *p* = 0.008), and familial schizophrenia history (OR = 0.250, *p* = 0.016) in comparison with the recovered group. Non-recovered participants were more likely to have lifetime alcohol abuse (OR = 0.381, *p* = 0.002) or addiction (OR = 0.351, *p* = 0.044), as well as cannabis abuse (OR = 0.480, *p* = 0.003) or addiction (OR = 0.449, *p* = 0.004) than recovered patients, while the differences between groups regarding rates for abuse or addiction to other substances were not significant. Non-recovered people had less insight at TIPP admission (OR = 1.852, *p* < 0.001) in contrast with recovered participants. Non-recovered patients reported worse functional level at baseline measured with GAF (OR = 1.045, *p* < 0.001) and SOFAS (OR = 1.046, *p* < 0.001) than recovered people. Non-recovered participants had higher illness severity rates at the TIPP admission (OR = 0.700, *p* < 0.001) in comparison with recovered people. Regarding the diagnosis at 3-year follow-up, the non-recovered group had more people with schizophreniform or brief psychosis (OR = 7.481, *p* < 0.001), and less people with bipolar disorder (OR = 4.525, *p* = 0.003) than the recovered group, as well as more non-recovered people having schizophrenia (68.4%) in comparison with the recovered group (47.8%).

**Table 2:** Sociodemographic and clinical data according to recovery (N = 325)

	Non-recovery 57.5% (*N* = 187)	Recovery 42.5% (*N* = 138)	Odds ratio	95% CI of OR		*p* value
				LCI	UCI	
Age in years, M (SD)	24.23 (4.64)	25.25 (4.93)	1.046	0.998	1.095	0.059
Sex, male, % (N)	72.2 (135)	63.0 (87)	1.522	0.950	2.437	0.081
DUP in days, Mdn (IQR)^a^	140.00 (653.00)	52.00 (231.50)	0.597	0.453	0.788	< 0.001
Number of hospitalizations, % (N)			0.529	0.397	0.704	< 0.001
None	19.3 (36)	31.9 (44)				
Single	26.7 (50)	41.3 (57)				
Multiple	54.0 (101)	26.8 (37)				
Socio-economical level, % (N)			1.077	0.802	1.448	0.621
Low	21.9 (41)	23.9 (33)				
Intermediate	46.5 (87)	38.4 (53)				
High	31.6 (59)	37.7 (52)				
Age of onset in years, M (SD)	22.55 (5.03)	24.17 (4.99)	1.067	1.020	1.116	0.005
Education in years, M (SD)	9.59 (2.42)	10.51 (2.83)	1.155	1.045	1.277	0.005
Marital status, % (N)						
Single	85.0 (153)	84.8 (117)	Ref.cat			
Married	8.9 (16)	10.1 (14)	1.144	0.537	2.438	0.727
Divorced	5.0 (9)	1.4 (2)	0.291	0.062	1.370	0.118
Cohabitation	1.1 (2)	3.6 (5)	3.269	0.623	17.149	0.161
Professional activity, % (N)						
Unemployed	57.1 (105)	36.0 (49)	Ref.cat			
Full time job	7.6 (14)	13.2 (18)	2.755	1.268	5.989	0.011
Student/traineeship	10.9 (20)	22.8 (31)	3.321	1.723	6.404	< 0.001
Part time job	2.7 (5)	4.4 (6)	2.571	0.748	8.835	0.134
Disability annuity	1.6 (3)	2.9 (4)	2.857	0.616	13.259	0.180
On Sickness leave	20.1 (37)	20.6 (28)	1.622	0.893	2.945	0.112
Lifestyle, % (N)						
Family	16.9 (31)	26.7 (36)	Ref.cat			
Independent household	21.3 (39)	23.7 (32)	0.707	0.361	1.381	0.310
With friends	49.2 (90)	45.2 (61)	0.584	0.327	1.042	0.069
Pension/care home	4.9 (9)	1.5 (2)	0.191	0.038	0.953	0.044
Unsettled (hotel, shelter homeless)	7.7 (14)	3.0 (4)	0.246	0.073	0.826	0.023
Premorbid Adj. (PAS) M (SD)						
Childhood	0.34 (0.19)	0.26 (0.16)	0.099	0.024	0.403	0.001
Early adolescence	0.35 (0.18)	0.29 (0.16)	0.128	0.030	0.547	0.006
Social	0.31 (0.22)	0.25 (0.19)	0.258	0.078	0.858	0.027
Academic	0.39 (0.21)	0.31 (0.18)	0.141	0.041	0.485	0.002
Total	0.34 (0.18)	0.28 (0.14)	0.079	0.016	0.388	0.002
Past suicide attempt^b^, % (N)	17.0 (31)	9.6 (13)	0.519	0.260	1.035	0.063
History of trauma^c^, % (N)	35.8 (67)	26.8 (37)	0.656	0.406	1.061	0.086
Migration in adversity^d^, % (N)	32.1 (60)	25.4 (35)	0.719	0.440	1.176	0.189
Forensic history, % (N)	20.2 (34)	8.9 (10)	0.386	0.182	0.819	0.013
Criminal offences during program, % (N)	13.6 (14)	3.7 (4)	0.247	0.078	0.777	0.017
Psychiatric history^e^, % (N)	64.8 (118)	55.1 (75)	0.667	0.423	1.051	0.081
Familial psychiatric history, % (N)	62.4 (106)	47.0 (62)	0.535	0.337	0.848	0.008
Familial schizophrenia history, % (N)	24.5 (40)	13.2 (17)	0.467	0.250	0.870	0.016
Lifetime substance abuse^e^, % (N)						
Alcohol	27.6 (50)	12.7 (17)	0.381	0.208	0.697	0.002
Cannabis	41.0 (75)	25.0 (33)	0.480	0.293	0.785	0.003
Other substances	11.8 (22)	8.0 (11)	0.651	0.304	1.392	0.268
Lifetime substance addiction^e^, % (N)						
Alcohol	9.9 (18)	3.7 (5)	0.351	0.127	0.971	0.044
Cannabis	33.3 (61)	18.3 (24)	0.449	0.262	0.769	0.004
Other substances	8.1 (15)	3.6 (5)	0.432	0.153	1.218	0.113
Insight at presentation, % (N)			1.852	1.348	2.545	< 0.001
Absent	38.7 (70)	22.8 (31)				
Partial	45.9 (83)	45.6 (62)				
Complete	15.5 (28)	31.6 (43)				
GAF, M (SD)	36.68 (14.60)	47.74 (17.17)	1.045	1.028	1.061	< 0.001
SOFAS, M (SD)	38.44 (14.24)	48.67 (16.04)	1.046	1.029	1.063	< 0.001
CGI, M (SD)	4.89 (1.32)	4.21 (1.44)	0.700	0.581	0.842	< 0.001
Diagnostic^f^, % (N)						
Schizophrenia	68.4 (128)	47.8 (66)	Ref.cat			
Schizophreniform/brief	3.7 (7)	19.6 (27)	7.481	3.094	18.085	< 0.001
Schizo-affective	13.4 (25)	8.7 (12)	0.931	0.440	1.970	0.852
Major depression^g^	3.2 (6)	4.3 (6)	1.939	0.602	6.248	0.267
Bipolar disorder	3.2 (6)	10.1 (14)	4.525	1.662	12.318	0.003
Other	8.0 (15)	9.4 (13)	1.681	0.755	3.740	0.203

% percentage, *N* number of participants, *CI* confidence interval, *OR* odds ratio, *LCI* lower confidence intervals, *UCI* upper confidence intervals, *M* mean, *SD* standard deviation, *DUP* duration of untreated psychosis, *Mdn* median, *IQR* interquartile range, *Ref.cat* reference category, *Adj.* Adjustment, *PAS* Premorbid Adjustment Scale, *GAF* Global Assessment of Functioning, *SOFAS* Social and Occupational Functioning Assessment, *CGI* Clinical Global Impression

^a^Raw data are presented, however the test statistics were based on log10 (+ constant) transformed data because of extreme positive skewness; DUP was defined as the time between the onset of psychosis defined by the CAARMS instrument and the admission to the TIPP; ^b^listed using ICD-10 classification; ^c^experience one or more physical, emotional or sexual abuse prior the age of 16; ^d^migrate in adverse context, such as, seeking protection for political reasons, threat of death, exposure to war o extreme poverty, etc.; ^e^assessed according to DSM-IV criteria; ^f^diagnostic process is based on criteria from the DSM-IV, the diagnosis at 36-months of treatment was used; ^g^with psychotic features

The remaining variables (age, sex, socio-economical level, marital status, past suicide attempt, history of trauma, migration in adversity, and psychiatric history) did not differ between recovered and non-recovered patients as can be seen in Table 2.

### Psychopathological and functional trajectories during the 3-year follow-up

**Fig. 2:**
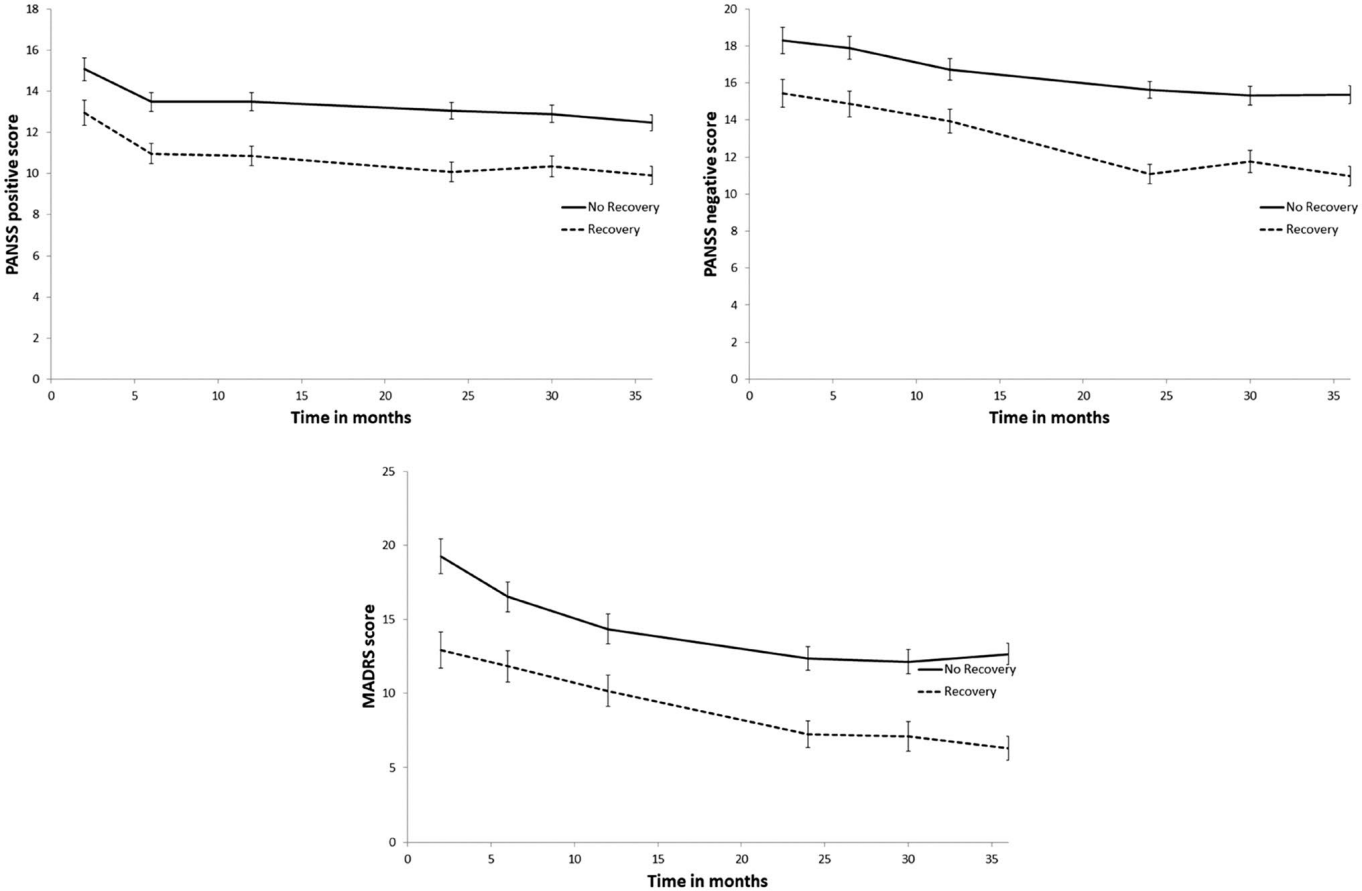
Symptomatology scores over 36 months of enrolment in the TIPP: comparison between non-recovered and recovered patients. *PANSS* Positive and Negative Syndrome Scale, *MADRS* Montgomery-Asberg Depression Rating Scale

**Fig. 3:**
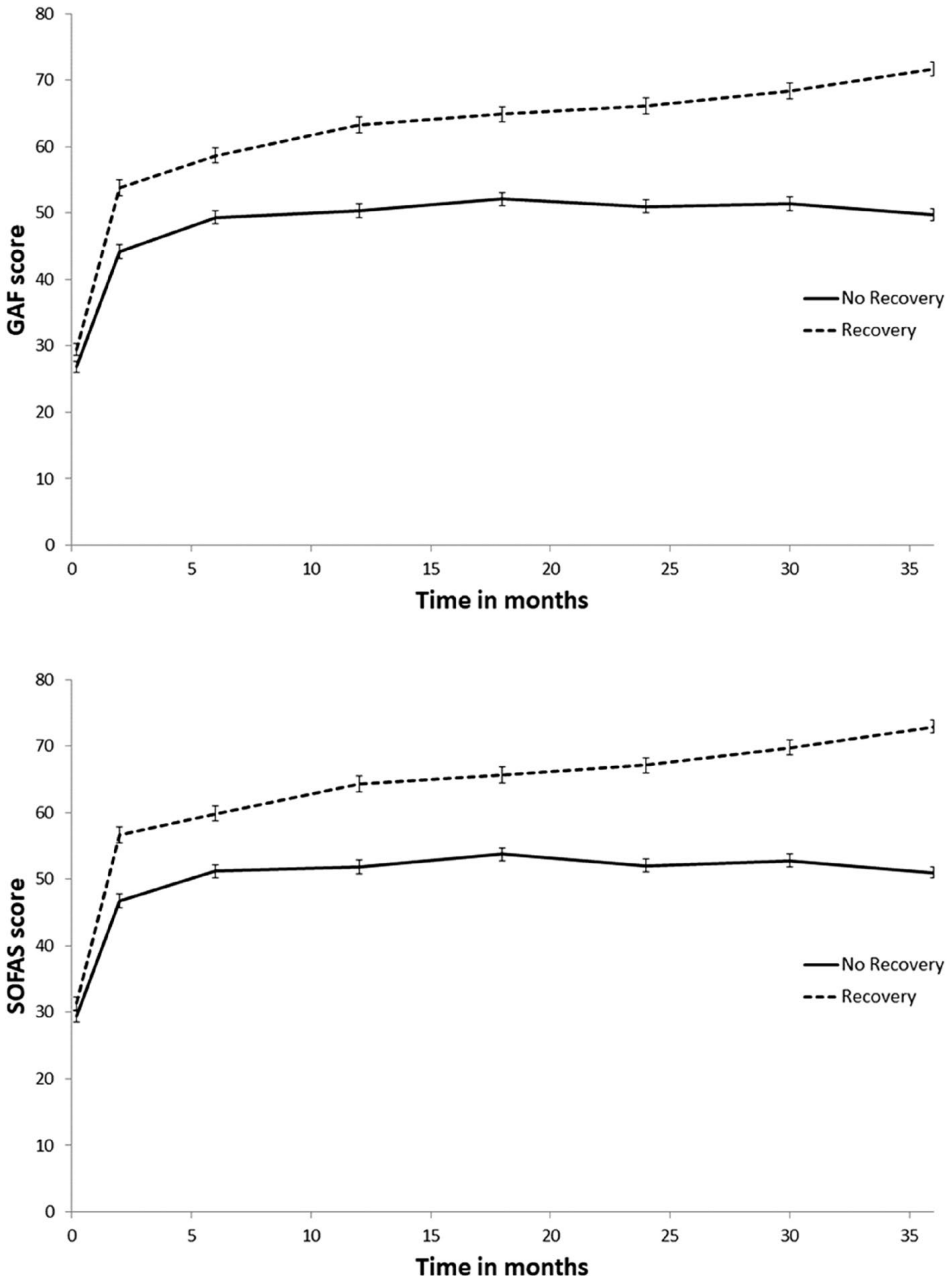
Functioning scores over 36 months of enrolment in the TIPP: comparison between non-recovered and recovered patients. *GAF* Global Assessment of Functioning, *SOFAS* Social and Occupational Functioning Assessment Scale

Results of longitudinal analyses through the entire follow-up in TIPP measured with the MMRM showed that the non-recovered and recovered groups of patients significantly differ regarding all the clinical and functional outcomes evaluated: PANSS positive symptomatology (*F*_1,250.375_ = 33,607, *p* < 0.001, mean difference = − 2.499, within-subject covariance = heterogenous Toeplitz), PANSS negative symptomatology (*F*_1,228,365_ = 45,910, *p* < 0.001, mean difference = − 3.666, within-subject covariance = heterogenous Toeplitz), depression symptomatology assessed with the MADRS (*F*_1,244.385_ = 39,071, *p* < 0.001, mean difference = − 5.398, within-subject covariance = heterogenous Toeplitz), and functioning assessed with the GAF (*F*_1,323.500_ = 153,418, *p* < 0.001, mean difference = 12.656, within-subject covariance = unstructured), and the SOFAS (*F*_1,323.356_ = 141,395, *p* < 0.001, mean difference = 12.341, within-subject covariance = unstructured) (see Figs. 2, 3). For all variables, symptoms trajectories differed significantly from the first assessment available with the exception of the SOFAS where the groups did not differ at baseline (*p* = 0.126) but differed significantly after 2-month follow-up (*p* < 0.001) and for all consecutive assessments. These results further strengthen the validity of the LCA classifications into recovered and non-recovered patients.

## Discussion

The purpose of this study was to explore which premorbid and baseline sociodemographic and clinical characteristics are associated with a good global recovery in patients with FEP at 3-year follow-up, by using a multimodal measure of recovery including clinical, functional and contextual information based on a Latent Class Analysis (LCA). To the best of our knowledge, this is the first study examining predictors of recovery at 3-year follow-up using a multimodal measure of recovery using LCA.

### The novel definition of the outcome in patients with FEP at 3-year follow-up

First, we highlight the novelty of the global multivariate measure of positive outcomes utilized with the multimodal LCA model. This strategy, was based on six binary variables used to identify latent classes, enabling us to overcome the lack of consensus about clinical recovery, considering functioning, psychosocial and contextual areas beyond symptomatic remission, and establish a more comprehensive definition of positive outcome [29]. After performing the LCA, we found that 42.5% of the whole sample recovered from the FEP at 3-year follow-up, a higher rate than previously found in meta-analysis with a shorter follow-up [41, 42]. Further research is needed to validate the global multivariate measure of positive outcomes, and to replicate the non-recovered and recovered groups and their trajectories. Moreover, these should be studied in juxtaposition with biomarkers and more variables, such as neurocognition, as well as the subjective aspects of the person in their daily life and sociocultural context, quality of life or life satisfaction.

### Risk and protective factors of poor outcome in relation to previous literature

Our results show that some premorbid and baseline sociodemographic and clinical characteristics differed between non-recovered and recovered groups. Regarding the variables widely known as risk variables for poor outcomes in patients with FEP, some of our results are in line with the existing literature. A low socio-economic status, a longer DUP, substance abuse and comorbid substance use disorders, a history of suicide attempts, family history of psychosis, and having non-affective psychosis, are associated with a worse outcome in patients with FEP [43–45].

We found that patients who failed to achieve recovery at 3-year follow-up had a longer DUP than the recovered participants, which is in line with previous literature including a recent meta-analysis on the topic [44, 46–50]. Having a shorter DUP is a PF against poor outcomes, this highlights the need for early detection of psychosis, and especially when the symptomatology appears it must be treated immediately. More programs are needed for early detection and also for the identification of vulnerable and high-risk people, this could allow them to be already linked in mental healthcare services and be able to treat them earlier.

Our findings revealed that more than half of the non-recovered people were hospitalized multiple times (two or more), while being admitted just once was more common in the recovered group. This suggests that being admitted just once may be protective and an indicator of a future better outcome; while being admitted recurrently is a reflect of a more severe form of the disorder but could also represent a risk factor for a poorer outcome [51]. This could be interpreted as a negative sequela that the negative impact that being admitted can have in patient’s self-stigma [52], and to the sometimes, traumatic experience related to being hospitalized [53]. More research investigating this more in depth is needed, exploring whether alternatives to readmission could be considered more often with crisis resolution and intensive home-based treatments as an alternative to rehospitalization.

Also, the evidence we obtained indicates that non-recovered patients at 36-months follow-up were younger at the onset of the psychosis and completed fewer years of education than the recovered ones. These results are in line with previous findings [54]. The earlier a psychosis starts, the more likely it is that the disease will cause disruption in their academic life, so they may complete fewer years of education. Additionally, having completed fewer years of education could indicate that clinical symptoms, interpersonal or cognitive difficulties lead to dropping out. Consequently, non-recovered people are less likely to be students and have higher unemployment rates, this was also reflected in our results. Previous studies observed that working is associated with recovery [42] and this is considered an important criterion to achieve functional recovery [55]. These low levels of employment since the onset highlight an ever-present problem in population with psychosis. Their occupational life is affected by the clinical, but also by socioeconomic factors [56]. A social and financial impoverishment may cause deterioration more than the disorder by itself, calling for better employment policies and early occupational programs [57]. What these indicate is the relevance of being enrolled in education or a job to achieve recovery. These could be considered a PF, people with FEP require to link themselves to occupational programs and also to educational projects adapted to their needs. This area needs to be studied more deeply in order to design realistic psychosocial interventions that can help them recover.

Our results show that the non-recovered patients reported deficits in premorbid adjustment more than the recovered people. Evidence considers a poor premorbid functioning as a proxy of a neurodevelopmental form of psychosis, usually associated with poorer outcomes [58]. These results are in line with previous studies, as better premorbid adjustment prior to the FEP onset has often been associated with recovery [50, 54, 55, 59, 60], and is a PF against poor outcomes. Early intervention preventive approaches are necessary, focusing on the premorbid period in childhood and adolescence as being a time where neurodevelopmental vulnerability is perceptible and the psychosocial and pharmacological treatments are effective [61].

Also, we found that the non-recovered patients showed poor social and academic functioning at baseline and during TIPP treatment. Additionally, our results show that preserved social functioning at the onset is related to recovery, which is in agreement with previous evidence [62]. Higher symptomatology severity rates were observed in the non-recovered group at baseline and throughout the entire TIPP follow-up. These results are in line with previous findings showing greater clinical severity at onset was a predictor of progression in psychosis [63]. Also, less acute symptoms at the onset were related to recovery [62]. Therefore, more functional deficits and acute symptoms at the onset of FEP may indicate a more severe course of psychosis, and a longer recovery context. Conversely, better functioning and milder symptoms are PF against poor outcomes.

In addition, in our sample sex did not differ between groups contrary to what previous studies observed [54, 55, 62].

In summary, the non-recovered patients in our sample had a more severe general profile at baseline, with poorer premorbid functioning and a longer DUP. More efforts to improve detection of these vulnerable group are needed and national efforts are being developed in order to improve the pathways to care in early intervention across Switzerland (https://psyyoung.ch/en/home/) (see recently published study protocol [64]). We hope that these efforts will improve the access to care of this vulnerable group.

Our findings expand knowledge on psychological resilience by detecting the variables associated with good recovery in FEP at 3-year follow-up. These could indicate potential resilient profiles, since previous studies suggested that better outcomes in psychosis are associated with internal protective factors such as greater resilience [65–68]. The premorbid and baseline sociodemographic and clinical characteristics that were associated with recovery, and that were identified as protective factors, may decrease the psychological vulnerabilities and may even enhance further psychological growth beyond recovery. Moreover, people who are on the road to recovery from FEP may know that they are improving because they receive feedback, and it may also be that this same process is promoting their resilience [69]. It could be about recovering and even more, rebuilding and growing after the episode. Furthermore, detecting the less favorable factors can allow designing specific interventions targeting modifiable variables, and otherwise carry out prevention. Future research should study the mechanisms behind resilience in different recovery status.

In this study, we also compared symptomatic and functional trajectories over 3 years between non-recovered and recovered patients in order to highlight when good and bad outcomes become evident. The analysis of clinical and functional longitudinal data revealed (not surprisingly given it included measures that were part of the multimodal set of clinical, functional and contextual recovery outcomes), differences between groups regarding PANSS symptoms, GAF, and SOFAS scores throughout study follow-ups. These results indicate the different recovery trajectories between groups from the FEP onset and emphasize the importance of early interventions, focusing on reducing the severity of symptoms and improving functioning of patients who have more unfavorable premorbid and baseline sociodemographic and clinical characteristics. A better understanding of recovery trajectories may enable a timely identification of patients’ needs and related choice of specific interventions, based on the assumption that recovery is both, a process and an outcome [70]. Also, beyond the link between the two groups in line to risk and PF, the clear distinction in trajectories and profile at baseline between the two groups also may indicate the presence of two different disease phenotypes with specific needs and levels of disease severity. It would be important to explore such groups in terms of biological markers in order to understand which endophenotypes may be operating as underlying mechanisms. Moreover, prognostic machine learning approaches combining such biological data with the demographic and clinical factors observed in the current study would help us predicting such evolution when pastiest access our services at onset.

### Limitations

While our study demonstrates some strengths (a large sample and also the use of LCA as an accurate and sensitive model-based technique to identify the best model with the most suitable latent classes), it also has some limitations. For example, data was available only for those who completed 3-year follow-up. However, those results can usefully be completed by a study on who disengaged early and whether there is a correlation with some onset variables. Given that in a certain proportion of cases the psychosis onset is before 18 years old, our results should be interpreted with caution for younger people with early onset of psychosis. A replication of our study in people younger than 18 years can help shed light on recovery trajectories and PF. Our study is based on a selection of six meaningful outcome criterion and could, of course, include other variables for classification. We are nevertheless confident that this should have a rather limited impact on our results: the classification is likely to be relatively robust to slight changes in indicators. Indeed, a patient does not need to achieve every outcome criterion to be included in the recovery class. Rather this class is defined as a high probability (> 70%) to have achieved the different criterion. This allows to define recovery in a more individualized way based on the overall criterion pattern.

### Conclusions

Our findings suggest that some premorbid and baseline sociodemographic and clinical characteristics were associated with recovery, and protective factors against poor outcomes were a short DUP, being hospitalized just once, having completed more years of education, a better premorbid adjustment, a good functioning at the treatment onset, and less symptoms severity. In doing so, our results provide empirical data supporting previous constructs based on available literature. Considering this, particular attention should be paid to these characteristics when patients join programs of treatment. The early detection of patients with unfavorable premorbid and baseline sociodemographic and clinical characteristics is essential in determining the need for specific interventions. This requires further research to help design and develop new interventions that can be used from the onset of the disorder.

## Data Availability

The dataset of this study is available on request from the corresponding author.
